# A Data-Centric Approach to improve performance of deep learning models

**DOI:** 10.1038/s41598-024-73643-x

**Published:** 2024-09-27

**Authors:** Nikita Bhatt, Nirav Bhatt, Purvi Prajapati, Vishal Sorathiya, Samah Alshathri, Walid El-Shafai

**Affiliations:** 1 Department of Computer Engineering, U & P U. Patel, CSPIT, CHARUSAT, Changa, Gujarat India; 2 Department of Artificial Intelligence and Machine Learning, CSPIT, CHARUSAT, Changa, Gujarat India; 3 Smt. K. D. Patel Department of Information Technology, CSPIT, CHARUSAT, Changa, Gujarat India; 4 Faculty of Engineering and Technology, Parul Institute of Engineering and Technology, Parul University, Vadodara, Gujarat India; 5 Department of Information Technology, College of Computer and Information Sciences, Princess Nourah bint Abdulrahman University, P.O. Box 84428, Riyadh, 11671 Saudi Arabia; 6 Security Engineering Lab, Computer Science Department, Prince Sultan University, Riyadh, 11586 Saudi Arabia; 7 Department of Electronics and Electrical Communications Engineering, Faculty of Electronic Engineering, Menoufia University, Menouf, 32952 Egypt

**Keywords:** Deep learning, Model Centric Approach, Hyper parameter tuning, Data Centric Approach, Computer science, Scientific data

## Abstract

The Artificial Intelligence has evolved and is now associated with Deep Learning, driven by availability of vast amount of data and computing power. Traditionally, researchers have adopted a Model-Centric Approach, focusing on developing new algorithms and models to enhance performance without altering the underlying data. However, Andrew Ng, a prominent figure in the AI community, has recently emphasized on better (quality) data rather than better models, which has given birth to Data Centric Approach, also known as Data Oriented technique. The transition from model oriented to data oriented approach has rapidly gained momentum within the realm of deep learning. Despite its promise, the Data-Centric Approach faces several challenges, including (a) generating high-quality data, (b) ensuring data privacy, and (c) addressing biases to achieve fairness in datasets. Currently, there has been limited effort in preparing quality data. Our work aims to address this gap by focusing on the generation of high-quality data through methods such as data augmentation, multi-stage hashing to eliminate duplicate instances, to detect and correct noisy labels, using confident learning. The experiments on popular datasets, namely MNIST, Fashion MNIST, and CIFAR-10 were performed by utilizing ResNet-18 as the common framework followed by both Model Centric and Data Centric Approach. Comparative performance analysis revealed that the Data Centric Approach consistently outperformed the Model Centric Approach by a relative margin of at least 3%. This finding highlights the potential for further exploration and adoption of the Data-Centric Approach in various domains such as healthcare, finance, education, and entertainment, where the quality of data could significantly enhance the performance.

## Introduction

**Fig. 1:**
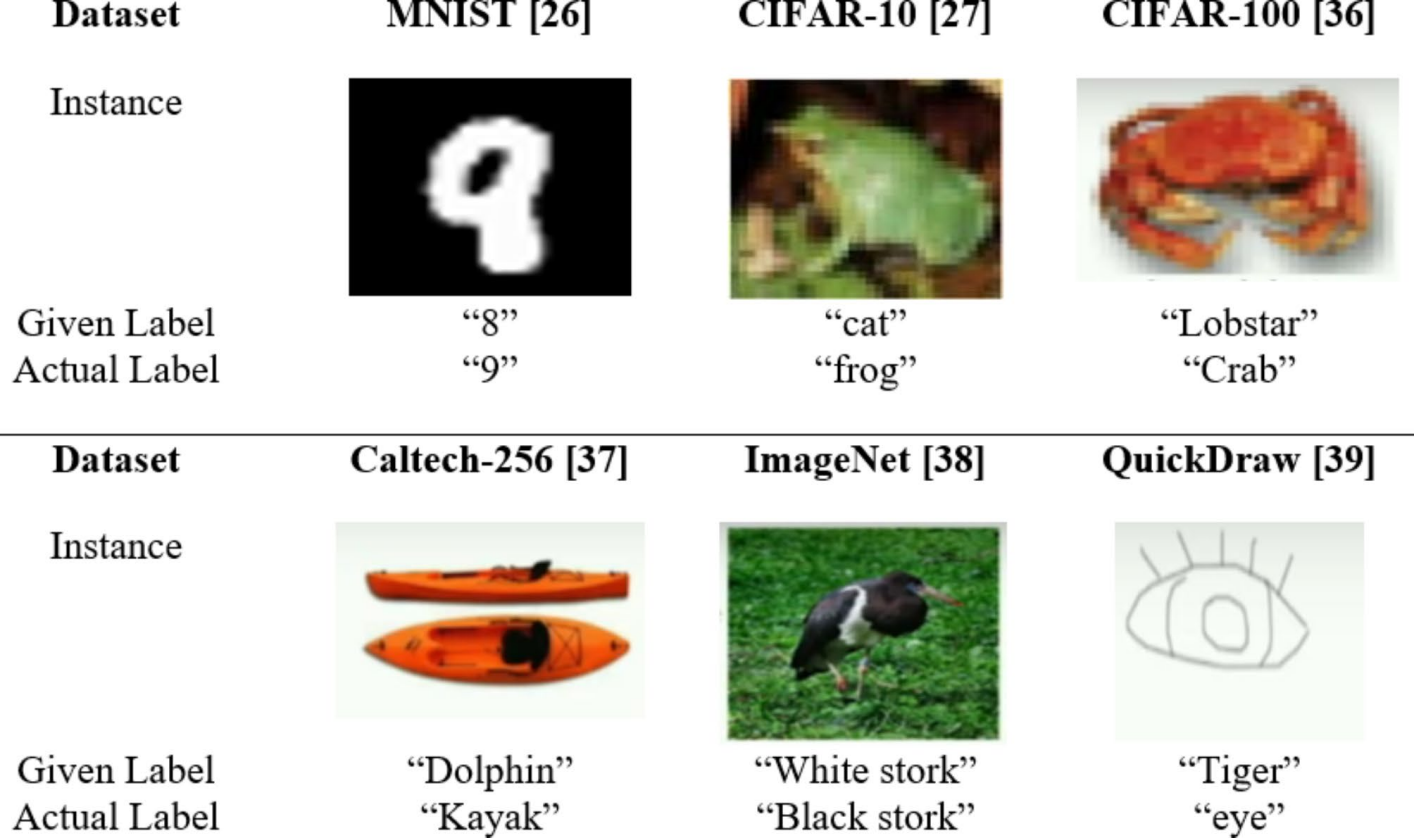
Example of Label Errors. A gallery of label errors images, is available at https://labelerrors.com. The image is created by the standard Powerpoint software with version 2016, URL: https://www.microsoft.com/en-in/microsoft-365/previous-versions/microsoft-office-2016.

The field of Artificial Intelligence (AI) has undergone a remarkable transformation due to two key factors: the abundance of data and the ever-increasing computational capabilities. Deep Learning, a subfield of AI has neural networks with multiple layers, which have shown incredible prowess in solving complex problems. The success of deep learning depends on huge amount of data, which is generated from a wide range of sources including social media, sensors, e-commerce, and more. Further, Deep Learning models are hungry for computational resources, and the advent of powerful GPUs. From the last few years, researchers have adopted Model Centric Approach to solve the complex problems. The objective of Model Centric Approach is to create new algorithms/ models and enhance performance of models by fine-tuning hyper parameters, without making changes to the existing data^40^. However, Quality data, as well as the sheer quantity of data, contributes to the enhanced performance of deep models – a new shift called Data Centric Approach. This approach recognizes the inherent value of human involvement in complex processes while harnessing AI serves as an adjunctive tool rather than a substitute for human expertise. Notably, Andrew Ng, a prominent figure in the AI community, has recently emphasized the importance of Data Centric Approach, advocating for a shift in focus from constantly improving AI models to instead prioritizing the improvement of the underlying data^15–17^. The data-centric approach presents numerous challenges such as (a) generation of quality data, (b) providing security and privacy to data, and (c) addressing biases for fairness within datasets^2,3,5^. Further, we observe that well-known datasets like MNIST^26^, CIFAR-10^27^, CIFAR-100^36^, Caltech-256^37^, ImageNet^38^, QuickDraw^39^ and many more contain noisy labels as shown in Fig. 1, where the actual label differs from the labels assigned to the dataset instances. If the frequency of noisy labels increases in the dataset, the performance of the model can be negatively affected as it trains with these noisy labels.

The primary aim of this research is to compare the effects of improved data versus enhanced models. While the Model-Centric Approach focuses on fine-tuning hyper parameters and algorithms, our research fills the gap by generating better data. We achieve this through data augmentation, multi-stage hashing to eliminate duplicates, and confident learning to detect and correct noisy labels. Using ResNet-18, we conduct experiments on datasets such as MNIST, Fashion MNIST, and CIFAR-10. Our results show that the Data-Centric Approach outperforms the Model-Centric Approach by at least 3%. We hypothesize that enhancing the quality of data through systematic methods like data augmentation, multi-stage hashing for eliminating duplicates, and confident learning for detecting and correcting noisy labels will significantly improve the performance of deep learning models, potentially outperforming the traditional Model-Centric approach.

The experiments using Model Oriented and Data Oriented approaches have adopted ResNet-18 due to capability of skip connections and global average pooling. The Model Centric Approach performs hyper parameter tuning to enhance model performance without altering the underlying data whereas Data Centric Approach increases the quality by (a) eliminating duplicate instances (b) detecting and correcting noisy labels and (c) data augmentation. The duplicate instances are eliminated using multi-stage hashing. The noisy labels are detected using confident learning and corrected by human annotation. The data augmentation involves rotation techniques. The experiments have been assessing to observe the impact of better model vs. better data.

Below are the contribution(s) of our work.

1. The proposed system generates quality data using Multi Stage Hashing. The Perceptual Hashing (pHash) is used to remove the duplicate images and CityHash function is used to speed up the processing.
2. The noisy labels are detected using confident learning. A threshold is optimized by conducting experiments on well-known datasets. The instances having probability distribution below optimized threshold value are considered as the noisy labels, which are corrected using human annotation.
3. The ResNet-18 model is used to evaluate performance of two approaches: Model-Centric and Data-Centric. To optimize performance of ResNet-18 in both approaches, automatisation of the hyperparameter tuning process using grid search is performed.
4. Experiments were conducted on well-known datasets to evaluate the impact of Data-Centric versus Model-Centric techniques. The experiments aimed to compare how these different approaches affect the overall model performance.

The rest of the paper is outlined as follows. Section 2 represents a background study and literature survey on Model Centric Approach and Data Centric Approach. Section 3 presents the proposed approach with the detailed discussion. Section 4 discusses experiments and results. The last section gives a conclusion with the future scope of the proposed approach.

## Background study

**Fig. 2:**
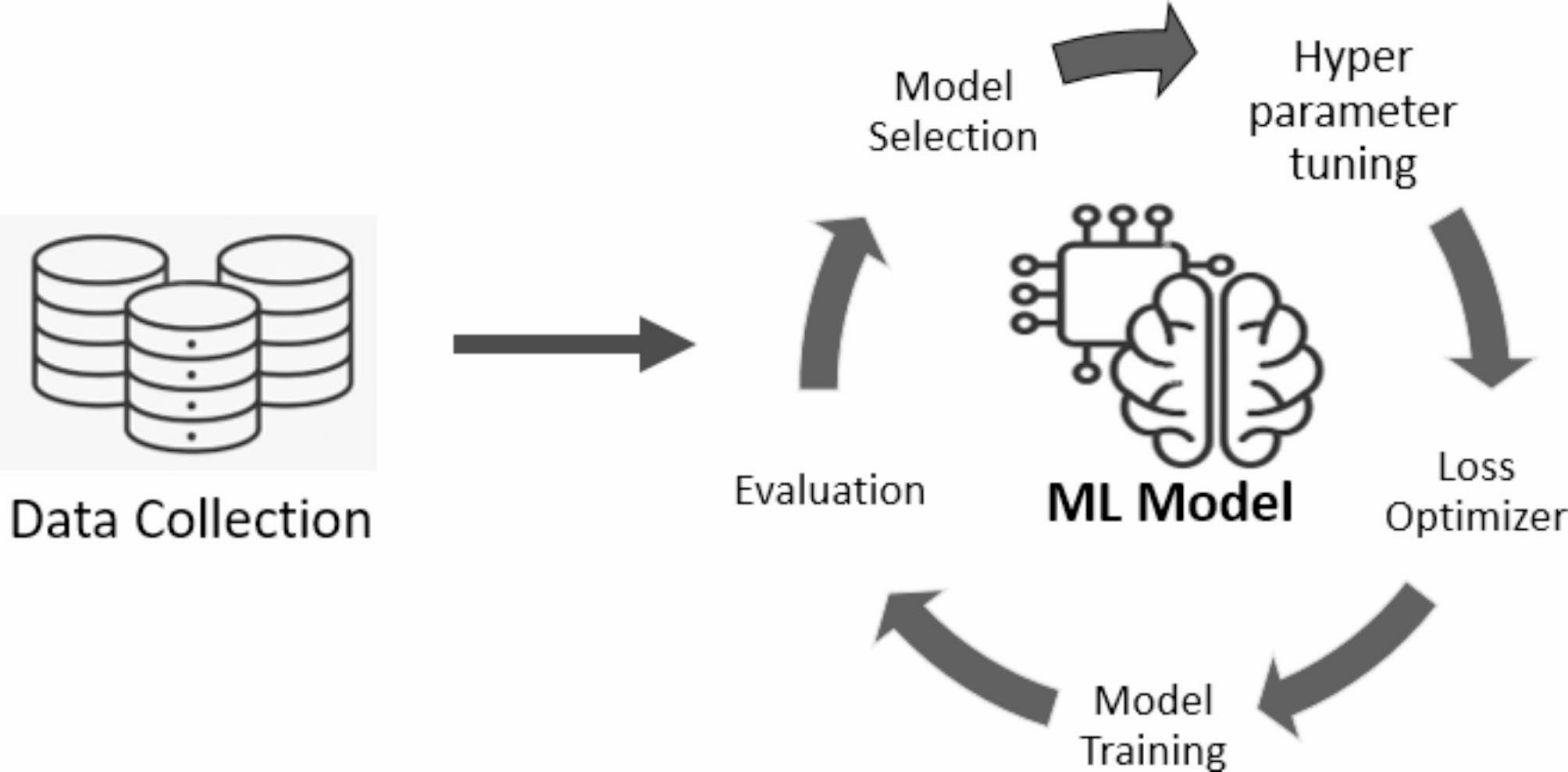
The steps for Model Centric Approach.

The conventional approach in AI research, known as the Model Centric Approach, has been a predominant strategy among researchers. This approach focuses on designing of novel algorithms or models to capture inherent patterns present within datasets^7,28,29,33,34,45^. Figure 2 provides an illustrative depiction of the key steps involved in the Model Centric Approach. This methodology has demonstrated significant success across diverse domains by leveraging renowned models such as AlexNet, VGGNet, GoogLeNet, and ResNet, among others^4,23^. These models have been pivotal in achieving remarkable performance benchmarks in various applications, showcasing their effectiveness in handling complex data structures and patterns. Their success has contributed substantially to the advancement and proliferation of AI technologies across multiple fields, underscoring their utility and significance within the Model Centric framework.

**Fig. 3:**
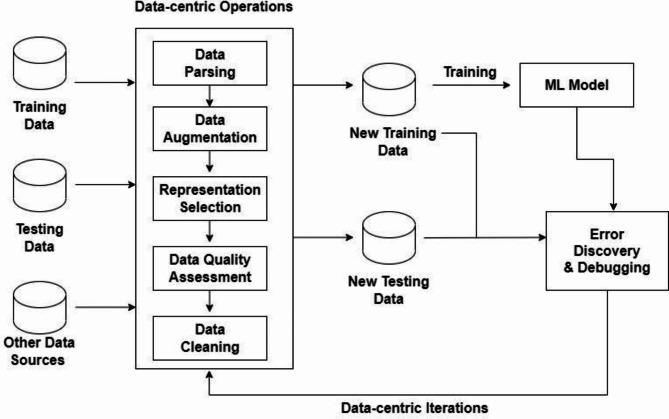
Operations performed by Data Centric Approach.

In real-world scenarios, the data used in the models contains inconsistencies, biases, noise, and missing values, posing challenges to the optimal performance of deep learning models^15–18^. Recognizing this, recent research endeavours have shifted their emphasis from solely designing models to prioritizing the generation of high-quality data to enhance the efficacy of deep models^1,2,4,12,30,40,45,46^. This shift characterizes the emergence of the Data Centric Approach, a methodology highlighted in Fig. 3 that encompasses various operations^12–15,21,24,31^ such as Data Parsing, Data Augmentation, Data Representation, Data Quality Assessment, and Data Cleaning.

The quality data is the central objective of the Data Centric Approach, which is one of the challenging issues for the researchers in AI domain. Table 1 provides the fundamental distinctions between these two approaches^16,19,32^.

**Table 1:** Key differences between Model Centric and Data Centric Approach.

Key Parameter	Model Centric Approach	Data Centric Approach
Focus	Builds complex models with a large number of parameters to fit the data.	Generates high quality data by applying different techniques.
Importance	Prioritizes model performance over data quality.	Prioritizes the quality and diversity of the data over model complexity.
Data set	This approach uses relatively small datasets, as large amounts of data can lead to slower training and model complexity.	This approach uses large datasets, as more data can improve model performance and reduce overfitting.
Efficiency	This approach can consume significant time and computational resources.	This approach can be more efficient and cost-effective, as better data quality can lead to higher performance with fewer resources.

The following section includes a literature review, an overview of the proposed system, and a discussion of experiments conducted on well-known datasets.

### Literature review

Recently, researchers have explored Data Centric Approach to generate quality data. The author in^22^ has combined “Convolutional Neural Network” (CNN) and “Recurrent Neural Network” (RNN) to capture the historical dependencies for financial time-series analysis. The CNN performs pre-processing task like “Image De-noising” that aims to reduce or eliminate noise from an image, enhancing its clarity and quality and RNN pre-processes sequence data. Further, CNNs can be also used to enhance the quality of data by data augmentation techniques like rotation, scaling and flipping. In^28,29^, the author outlined the factors that led to the transition from a model oriented to a data oriented approach and provided details on data oriented methods such as “Transfer Learning”, “Active Learning”, and “Semi-supervised Learning”. In addition, the difficulties encountered by each step—data preparation, cleaning, validation, and comprehension—are covered. In^35^, the author discussed multi-task learning (MTL) techniques, which by allowing parameters to be shared across many machine learning tasks, improves generalization and so represents a Data Centric approach. Attribute sharing allows one operation to use data from another, which improves the outcome in the end. It also helps with data enhancement. Author in^21^ compares the Model Centric and Data Centric Approaches to deep learning, with a particular focus on the role of data augmentation in improving model performance. The authors argue that the Data Centric Approach focuses on the critical role played by well-constructed and varied training datasets, is essential for attaining cutting-edge performance in deep learning applications. The author in^18^ proposes a Data Centric approach for ML that places greater emphasis on data quality and it is well-suited to large-scale ML systems, where data is often spread across multiple sources and may be noisy or incomplete. The author in^20^ provides an overview of the Data Centric Approach to ML, including data pre-processing, feature selection, and data augmentation techniques. The authors argue that the Data Centric Approach is particularly well-suited for handling large and complex datasets, which is helpful to improve the robustness and generalization of ML models.

In recent studies focusing on pose estimation utilizing a Data-Centric Approach, the algorithm undergoes training in two stages to accommodate resource limitations: (a) feature extraction and (b) temporal fusion across the video sequence. However, the absence of temporal context during encoder training emphasizes the need for enhancing data quality. The author in^6^ generates quality data using dense phase and segmentation annotation methods for model development. The dense phase involves annotating data in detailed manner and segmentation annotation involves labelling specific regions within the image. The detailed annotation helps to understand the boundaries in the data leading to improved quality of data. In^8^, the author employs a Data Centric Approach to perform Cassava Leaf Disease Classification, which classifies different types of diseases affecting cassava leaves. The quality data is generated by correcting mislabelled instances. In^9^, data quality is generated using the gradient clipping method, widely used for optimization. The method involves limiting or scaling the gradients of the model’s parameters during the training process to prevent them from becoming too large, which can lead to issues like exploding gradients and unstable training. By using gradient clipping, the author ensures that the data used for training machine learning models is of high quality and that the training process is stable and effective, leading to better model performance and results. In^10^, the author employs the neural backpropagation concept to identify noise labels present in the dataset. The neural backpropagation refers to the process of computing gradients of the model’s loss function with respect to its parameters. These gradients are used to update the model’s parameters during training, making it more accurate in its predictions. The author in^11^ focuses on enhancing the quality of data for the MNIST dataset by using data augmentation methods. These methods create diverse variations of the dataset to improve model training. The performance of different approaches is evaluated using the cross-entropy loss, that is extensively used measure for classification tasks, ultimately contributing to the understanding and comparison of various methods for digit recognition. The author in^43^ explores how adding causal probabilistic variables to data analysis models can improve prediction accuracy by leveraging causal relationships in the data. In^44^, author describes a strategy for placing data based on user movement in a hybrid cloud/edge system and uses a deep learning network to optimize data placement, enhancing efficiency and performance.

The distinction between the two approaches often comes down to prioritization rather than exclusivity—data-centric approaches prioritize improving data quality, while model-centric approaches focus on experimenting with models and algorithms. While combining both data pre-processing and model tuning is ideal, practical constraints such as time, computational resources, and expertise may require prioritizing one over the other. For example, if a quick deployment is needed, focusing on data quality might provide a faster route to a reliable model than extensive model tuning. Below section covers our methodology used to generate quality data followed result discussion and comparison between better model with better data.

## Proposed system

**Fig. 4:**
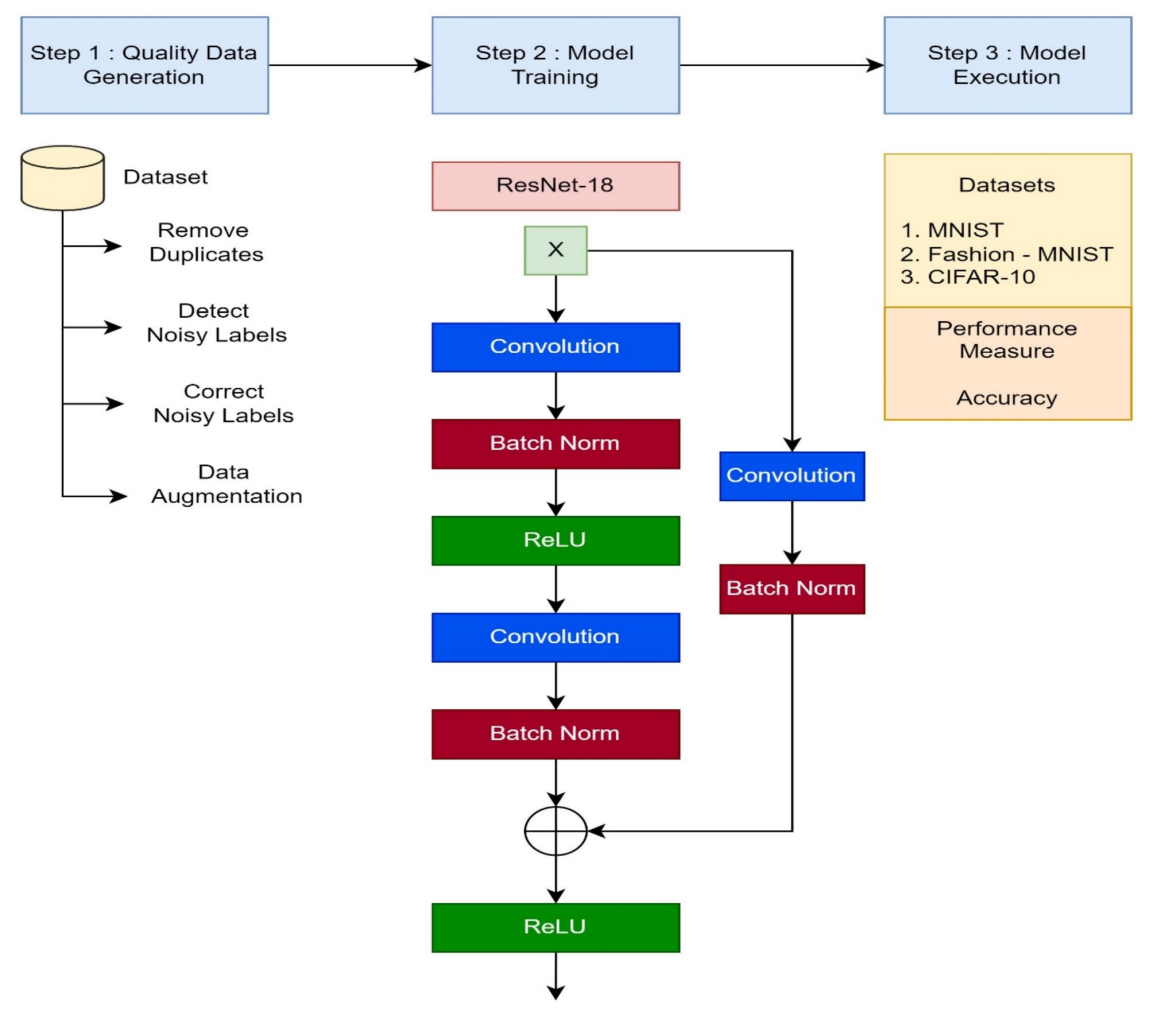
Flow of the proposed System.

The proposed system is divided into three Sect. (1) Generation of quality data (2) Training of the Model (3) Evaluation of the model. The step-by-step working of the proposed system is shown in the Figure-4.

Below section covers detail of each section.

**Step 1: Quality data generation**.

The proposed system has adopted multiple hash functions in stages to generate quality data. It has adopted two hash functions: Perceptual Hashing (pHash) and CityHash. The pHash function is used to identify duplicate images from the dataset. The CityHash function is used to speed up the processing time and also responsible to minimize the collision. The pHash function first converts image into grayscale to simplify the processing. The grayscale image undergoes Discrete Cosine Transform (DCT), which breaks image into frequency components in the form of matrix. The resulting values represent different frequencies in the image. The top-left corner of the transformed matrix contains the low-frequency components, which represent the most significant visual information, such as shapes and patterns. These components are crucial because they are less sensitive to changes like noise or minor alterations in the image, making them reliable for comparison and duplicate detection. To generate a perceptual hash, the system calculates the average value of low-frequency components. Each component is then compared to the average value. A binary value of ‘1’ is assigned if the component’s value is above the average and a binary value of ‘0’ is assigned if the component’s value is below the average. The duplicate images are identified by comparing their binary codes, which are removed from further processing. Another hash function, called CityHash, is used for tasks that require fast processing. These hash functions distribute data across the hash space and reduces the likelihood of collisions by producing the same hash value by spreading data across the hash space^41,42^.

The next step to generate quality data is to detect and correct noisy label using confident learning. The confident learning is used to utilize the predictions of a model to identify instances where the true label may be uncertain or incorrect. The process involves checking how confident the model is in its predictions and comparing these to the true labels. If the model’s confidence is low, it indicates a potential label error, which can then be corrected by a human annotator.

**Label noise detection using confident learning**.

Below section covers and notations of confident learning to detect the label noise.

**Notations of confident learning**.

$\:\stackrel{\sim}{y}$ : observed or noisy label

$\:{y}^{*}$: true or unobserved label

$\:{X}_{\stackrel{\sim}{y}=i,{y}^{*}=j\:}$: set of examples where the observed label is i but the true label is j.

$\:\text{P}(\stackrel{\sim}{\text{y}}=\text{i}|{\text{y}}^{\text{*}}=\text{j})$: transition probability that label j is misclassified as label i

Below are the steps followed by confident learning to detect the noise labels.

(i) Determine the threshold for each class as a substitution for machine confidence as shown in Eq. (1). The threshold$\:\:{\mathbf{t}}_{\mathbf{j}}$ for class j is computed by averaging the predicted probabilities for all samples with the observed label j. Cross-validation is used to train the model on the data, resulting in the determination of the threshold.
  1 $$\:{\mathbf{t}}_{\mathbf{j}}=\frac{1}{\left|{\mathbf{X}}_{\stackrel{\sim}{\mathbf{y}}=\mathbf{j}\:}\right|}\:\sum\:_{\mathbf{x}\in\:{\mathbf{X}}_{\stackrel{\sim}{\mathbf{y}}=\mathbf{j}}}\mathbf{P}\:\left(\stackrel{\sim}{\mathbf{y}}=\mathbf{j};\mathbf{x},\varvec{\uptheta\:}\right)$$

Where $\:\left|{\mathbf{X}}_{\stackrel{\sim}{\mathbf{y}}=\mathbf{j}\:}\right|$ is the number of samples in the dataset belonging to class j.

$\:\mathbf{P}\:\left(\stackrel{\sim}{\mathbf{y}}=\mathbf{j};\mathbf{x},\varvec{\uptheta\:}\right)$ represents the predicted probability that the model assigns to sample x belonging to class j, given the model parameters θ. The proposed system has adopted Random Forest method, which provides probability distribution over the possible labels for each instance in the labelled dataset.

(ii) The next step involves identifying label errors. The threshold $\:{\mathbf{t}}_{\mathbf{j}}$ is used to distinguish between predicted instances and those where the model’s prediction is uncertain as shown in Eq. (2).
  2 $$\:{\text{X}}_{\stackrel{\sim}{\text{y}}=\text{i},{\text{y}}^{\text{*}}=\text{j}\:}=\{\mathbf{x}\in\:{\mathbf{X}}_{\stackrel{\sim}{\mathbf{y}}=\mathbf{i}}\::\:\mathbf{P}\:\left(\stackrel{\sim}{\mathbf{y}}=\mathbf{j};\mathbf{x},\varvec{\uptheta\:}\right)\ge\:{\mathbf{t}}_{\mathbf{j}}\}$$

By choosing instances where $\:\mathbf{P}\:\left(\stackrel{\sim}{\mathbf{y}}=\mathbf{j};\mathbf{x},\varvec{\uptheta\:}\right)\ge\:{\mathbf{t}}_{\mathbf{j}}$, the method ensures that only those instances with a high likelihood of being mislabeled are flagged for further review. This helps in reducing the number of correctly labeled instances mistakenly flagged as mislabeled. These noisy labels are then reviewed by human annotators for correction.

The last step to generate quality data is using Data Augmentation. The experiments involve implementing random rotation with a 0.05 factor, random contrast adjustments with a 0.5 factor, and random translations within a range equivalent to 20% of the image dimensions.

**Step 2: Model training**.

The dataset is passed to the pretrained Residual network (ResNet-18). The key characteristics of ResNet-18: (a) skip connections, which specifies the difference between expected output and current output. By propagating the difference through network, ResNet-18 can train very deep network without the problem of vanishing gradient. (b) global average pooling, which averages the feature maps spatially to obtain a fixed-size vector for classification. This shrinks the amount of factors in the model and helps to prevent overfitting.

**Step 3: Model execution**.

The Model Centric and Data Centric Approach uses ResNet-18 as a baseline model to estimate the result. In the Model-Centric technique, the result of the model is assessed using original data, while in the Data-Centric Approach, the evaluation is based on quality data generated at the conclusion of step 1.

## Experimental set up and result discussion

The proposed system was evaluated on the well-known datasets like MNIST^26^, Fashion MNIST^25^, and CIFAR-10^27^. Table 2 represents dataset statistics. The MNIST^26^ dataset contains images of handwritten grayscale digits ranging from 0 to 9. The Fashion MNIST^25^ dataset consists of clothing items from 10 different categories like T-shirt, dress, sneaker, etc. The CIFAR-10^27^ contains 60,000 colourful images, with 6000 photos in each of the ten different categories including “airplanes”, “automobiles”, “birds”, “cats”, “deer”, “dogs”, “frogs”, “horses”, “ships”, and “trucks”.

**Table 2:** Dataset statistics.

	MNIST [26]	Fashion MNIST [25]	CIFAR-10 [27]
**Images**	70,000	70,000	60,000
**Image Size**	(28,28,1)	(28,28,1)	(32,32,3)
**Classes**	10	10	10

The experiments conducted on a system consisting of an Intel Core i7 8th generation processor, 32 GB of RAM, 2 TB hard disk, and NVIDIA CUDA cores with a count of 3840. Below section covers various experiments along with the conclusions.

**Experiment 1: Hyper parameter tuning**.

The hyper parameter is a fundamental aspect of both Model Centric and Data Centric Approach, which significantly influence the training process. Table 3 shows the hyper parameter settings used by both the approaches. There are various methods available to determine the optimal hyper parameters for a given model. In this work, grid search technique is used to find the best combination of hyper parameters that result in the highest model performance. The model is trained and evaluated on each combination of hyper parameters, and the set of hyper parameters that result in the best performance are selected as the optimal hyper parameters. Both approaches use the ResNet-18 model.

**Table 3:** Hyper parameter settings for Model Centric and Data Centric Approach.

	CIFAR-10	MNIST	Fashion MNIST
**batch_size**	32	4096	4096
**epochs**	5	5	10
**Dropout**	0.25	0.2	0.2
**Optimizer**	RMSprop	Adam	Adam
**Loss**	Cross entropy	Cross entropy	Cross entropy

**Conclusion 1**:

The results from Experiment 1 demonstrate that careful tuning of hyper parameters, including batch size, epochs, dropout rate, optimizer, and loss function, is essential for maximizing model performance. The use of grid search allowed for an exhaustive evaluation of possible configurations, leading to the selection of hyper parameters that yielded the best results. This approach ensures that the models are not only trained efficiently but also capable of generalizing well to new data. Therefore, both Model Centric and Data Centric approaches benefit significantly from optimized hyper parameter settings, as they directly impact the effectiveness and robustness of the models.

**Experiment 2: To fix the threshold for the detection of the noisy labels during data centric approach**.

**Fig. 5:**
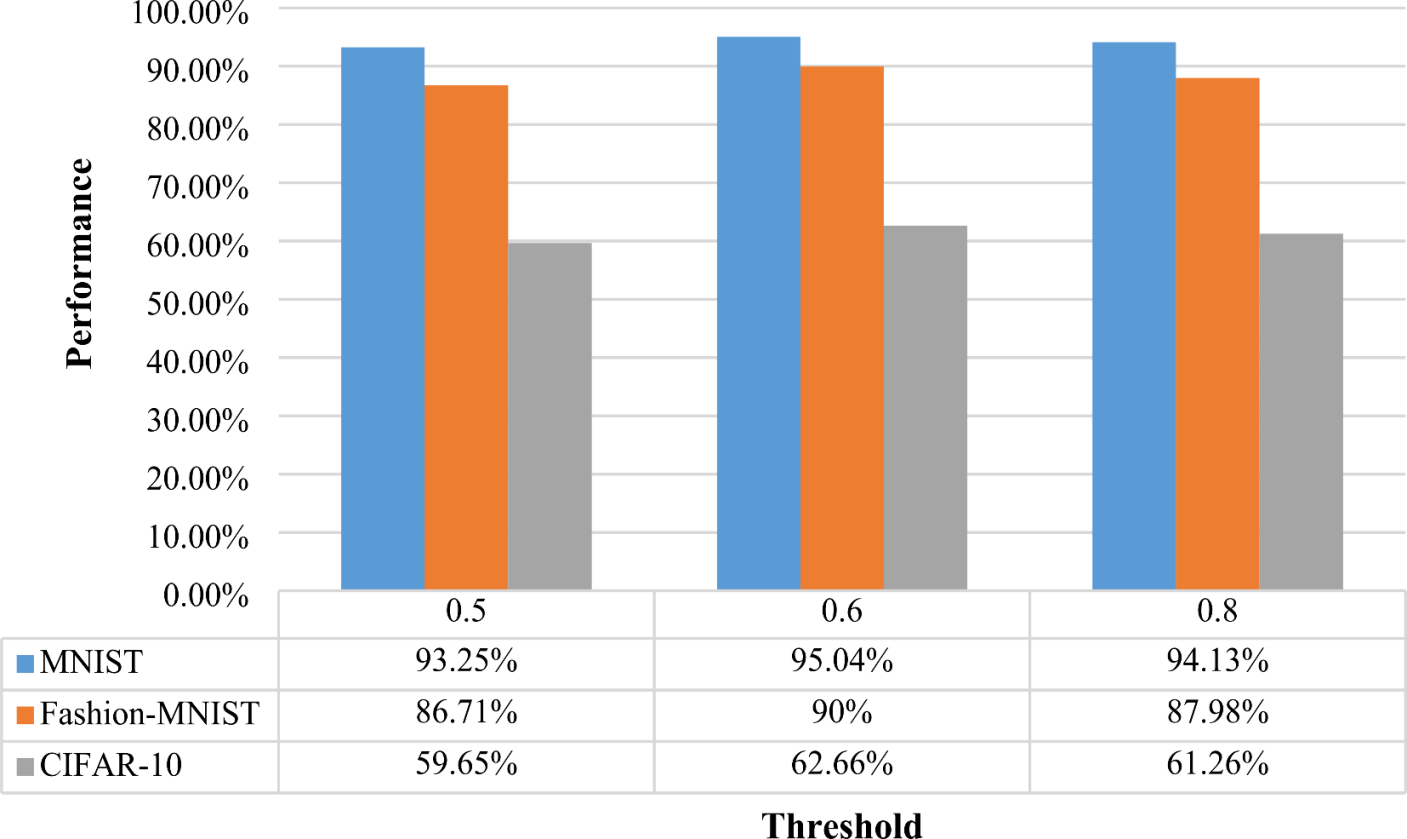
Different threshold value for the detection of Noisy Labels.

The biggest challenge during Data Centric Approach is to find out the noisy labels. The Random Forest generates probability for each class. The noisy labels from the dataset are detected from the threshold value. The instances having probability distribution below optimized threshold value are considered as the noisy labels. Experiments are performed with different threshold values as shown in Fig. 5. The result shows that a threshold value 0.6 leads to the superior performance on all the datasets. So, it is selected for the other experiments.

**Conclusion 2**:

The objective of the experiment is to find the best threshold value to differentiate noisy labels from clean ones. After testing various thresholds, a value of 0.6 was found to consistently perform the best across all datasets. This threshold was chosen for further experiments because it effectively identifies noisy labels while preserving the accuracy of the clean labels, thus improving data quality without reducing model accuracy.

**Experiment 3: To compare performance of model centric and data centric approach**.

**Fig. 6:**
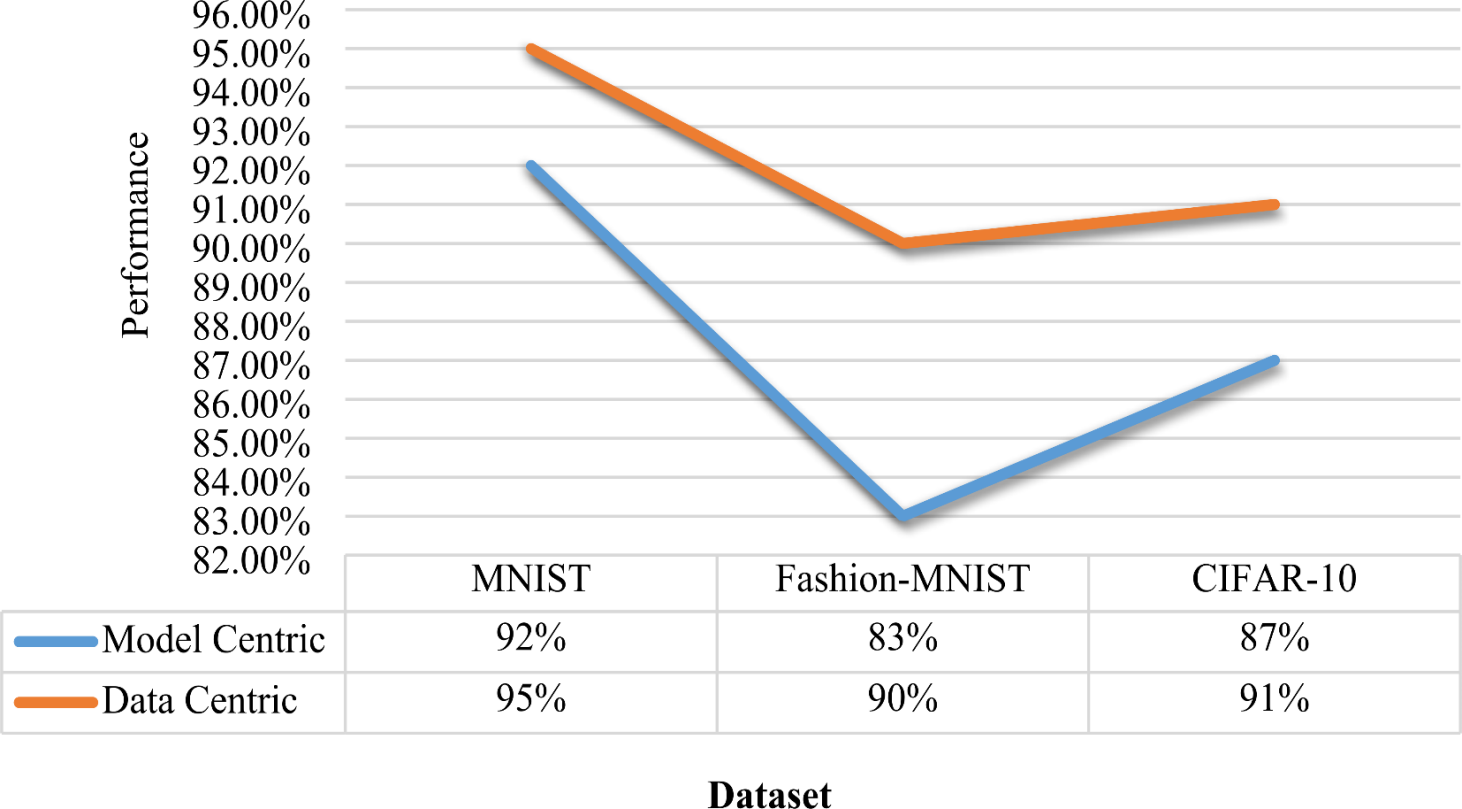
Performance Comparison of Model Centric and Data Centric Approach.

All the instances having value less than the threshold value is considered to have the noisy labels. To generate the quality data during Data Centric Approach, these instances are provided to the human annotator and labels are updated. The model was retrained with corrected labels to assess the influence of quality data. Figure 6 illustrates the performance comparison between the Model-Centric and Data-Centric Approaches across all three datasets.

Above experiment demonstrates a positive correlation between input data quality and model performance. One can generate the quality data and can increase the result of the system. Apart from algorithm design and hyper parameter tuning, the impact of data quality significantly affects system performance. This finding has significant implications for improving the accuracy and effectiveness of ML models, especially in scenarios where data quality is a critical factor in determining model performance. The experiment concludes that Data Centric Approach achieves better performance with a relative margin of at least 3% than Model Centric Approach.

**Conclusion 3**:

The experiment shows that besides optimizing algorithms and hyper parameters, it is essential to focus on data quality. Clean and accurate data reduce noise and bias, helping the model learn better and perform well on new data. Therefore, improving data quality can lead to more reliable and robust machine learning systems, especially in situations where data quality is crucial.

**Experiment 4: To observe performance of model centric and data centric approach with various dataset size**.

Table 4 illustrates the performance of two approaches on MNIST dataset with different training, validation, and testing size. It also presents the corresponding accuracy values achieved during these stages.

**Table 4:** Impact of model centric and data centric approach on MNIST dataset.

Round	Dataset Size			Accuracy (Model Centric)			Accuracy (Data Centric)		
	Train	Validation	Test	Train	Validation	Test	Train	Validation	Test
1	2000	500	3500	86%	73%	78%	87%	81%	83%
2	3000	900	3500	88%	75%	82%	90%	84%	85%
3	5000	1200	3500	91%	78%	85%	93%	88%	89%
4	6000	1500	3500	93%	80%	86%	96%	90%	91%
5	8000	2000	3500	94%	83%	92%	100%	91%	95%

Similarly, Table 5 illustrates the performance of two approaches on Fashion MNIST dataset with different training, validation, and testing size. It also presents the corresponding accuracy values achieved during these stages.

**Table 5:** Impact of model centric and data centric approach on fashion MNIST dataset.

Round	Dataset Size			Accuracy (Model Centric)			Accuracy (Data Centric)		
	Train	Validation	Test	Train	Validation	Test	Train	Validation	Test
1	1500	500	3000	83%	68%	73%	85%	83%	81%
2	2000	800	3000	87%	69%	76%	91%	86%	82%
3	4000	1500	3000	89%	81%	79%	92%	86%	84%
4	8000	2000	3000	90%	82%	81%	93%	90%	86%
5	10,000	4000	3000	91%	84%	83%	97%	91%	90%

Table 6 illustrates the performance of two approaches on CIFAR-10 dataset with different training, validation, and testing size. It also presents the corresponding accuracy values achieved during these stages.

**Table 6:** Impact of model centric and data centric approach on CIFAR-10 dataset.

Round	Dataset Size			Accuracy (Model Centric)			Accuracy (Data Centric)		
	Train	Validation	Test	Train	Validation	Test	Train	Validation	Test
1	4000	1000	2000	79%	67%	72%	81%	69%	76%
2	5000	2000	2000	81%	69%	74%	83%	73%	79%
3	7000	2500	2000	83%	72%	76%	84%	75%	80%
4	9000	3000	2000	84%	75%	80%	86%	79%	81%
5	12,000	4000	2000	86%	78%	87%	92%	82%	91%

**Conclusion 4**:

1. In each round, the Data Centric Approach consistently outperforms the Model Centric Approach in terms of accuracy. This indicates that the Data Centric Approach, which utilizes a pre-processed dataset, is more effective in achieving higher accuracy.
2. Both approaches generally perform well during training and testing. However, Data Centric Approach consistently achieves higher validation accuracy compared to the Model Centric Approach. This suggests that the Data Centric Approach is better at generalizing to unseen data.
3. In Round 5, the Model Centric Approach achieves 100% accuracy on the training data but a lower accuracy on the validation and testing datasets. This is a classic sign of overfitting, where the model has learned the training data too well and struggles to generalize to new data.
4. The Data Centric Approach demonstrates more consistent improvements in accuracy across the rounds, indicating that it is less sensitive to changes in dataset size.

## Conclusion

This research has highlighted the paramount importance of data quality in achieving superior performance for modern ML algorithms. The primary focus of this work has been to generate high-quality data using confident learning, which proves to be an essential factor in enhancing model effectiveness. By carefully addressing the issue of noisy labels, we have paved the way for leveraging quality data as input to the model. The evaluation of model performance clearly demonstrates the advantages of utilizing high-quality data over the traditional Model Centric Approach, which tends to concentrate on optimizing model architecture and parameters. Our findings confirm that “Data Quality” perform a critical role in the development of effective ML models, and dedicating efforts to generate such high-quality data yields substantial improvements in overall model performance. As we navigate through the ever-evolving landscape of ML, it is evident that Data Centric practices are gaining traction and driving new advancements in the field. Our work reinforces the notion that the foundation of any successful ML endeavor lies in the quality of data used, and striving for excellence in data quality holds the key to unlocking the full potential of machine learning algorithms across diverse applications and domains.

## Data Availability

The datasets used and analysed during the current study available from the corresponding author on reasonable request.
